# What is the structural chemistry of the living organism at its temperature and pressure?

**DOI:** 10.1107/S2059798320000546

**Published:** 2020-02-06

**Authors:** John R. Helliwell

**Affiliations:** a Department of Chemistry, University of Manchester, Manchester M13 9PL, England

**Keywords:** structural biology, X-rays, neutrons, electrons

## Abstract

The three probes of the structure of matter (X-rays, neutrons and electrons) in biology have complementary properties and strengths. The balance between these three probes within their strengths and weaknesses is perceived to change, even dramatically so at times. For the study of combined states of order and disorder, NMR crystallography is also applicable. Of course, to understand biological systems the required perspectives are surely physiologically relevant temperatures and relevant chemical conditions, as well as a minimal perturbation owing to the needs of the probe itself. These remain very tough challenges because, for example, cryoEM by its very nature will never be performed at room temperature, crystallization often requires nonphysiological chemical conditions, and X-rays and electrons cause beam damage. However, integrated structural biology techniques and functional assays provide a package towards physiological relevance of any given study. Reporting of protein crystal structures, and their associated database entries, could usefully indicate how close to the biological situation they are, as discussed in detail in this feature article.

## Abstract 2

The three probes of the structure of matter (X-rays, neutrons and electrons) in biology have complementary properties and strengths. For the study of combined states of order and disorder, NMR crystallography is also applicable. Overall, to understand a biological system, the requirements are physiologically relevant results. Reporting of protein crystal structures, and their associated database entries, could usefully indicate how close to the biological situation they are.

## 1. Introduction   

Crystallography is a key player in structural biology today , as are the microscopies and spectroscopies. These latter two areas include cryo-electron microscopy (cryoEM) and nuclear magnetic resonance (NMR) spectrosocopy. CryoEM, in particular in recent years, has allowed studies at ‘atomic resolution’ of large complexes that will not crystallize.

Perceptual dramatic changes in the field of structural biology have centred on the wonderful change in capabilities given by, for example, femtosecond time-slice X-ray lasers, the anti-blurring compensations in cryoEM for improved resolution images of the noncrystallized sample state, the major improvements in capabilities for the experimental determination of protonation states using neutron crystallography and the use of fully tuneable synchrotron radiation for optimized anomalous dispersion applications, even on a microcrystal. For the study of combined states of order and disorder, NMR crystallography has arrived. It is the overriding concept of the ISDSB conferences, an inspirational idea of Japanese colleagues, to bring the various measurement probes of structural biology, with their complementarities and results from their use, together. A further concept of the ISDSB conferences is to bridge academic research and industrial research and applications, because our results offer important new opportunities for the treatment of diseases. The conference website adds further detail to this conference series concept (https://isdsb2019.symposium-hp.jp/about/overview/).

## 2. Some history   

A thorny issue, especially in the 1970s, was the relevance of the protein crystalline state to the solution state of a protein inside the biological cell. NMR provided atomically detailed results in solution, and of course protein crystallography provided atomic details for a protein in the solid state. I recall Professor R. J. P. Williams at an Oxford University Laboratory of Molecular Biophysics seminar explaining to us that the core aromatic side chains in a protein studied by NMR spectroscopy must be flipping on the NMR timescale, whereas a crystal structure of the same protein appeared to show static side chains, and presumably therefore that they were too tightly packed by the crystal to move. This was obvious when one considers that in myoglobin, for example, there is no way for the oxygen to get to the haem based on the crystal structure without some structural movements.

In fact a protein crystal’s solid state is a liquid-like state anyway, by which I mean that the solvent content is very significant and can vary from about 35% up to even 80%. Studies of the structure and function of an enzyme in the crystalline state were to my mind greatly facilitated by the invention of the flow cell (Wyckoff *et al.*, 1967 ▸). An example executing this to excellent effect was in the crystallographic studies of glycogen phosphorylase (Hajdu *et al.*, 1987 ▸). David Blow was a pioneer in enzyme crystallography, and in his late career overview ‘*So do we understand how enzymes work?*’ (Blow, 2000 ▸) he lamented that a prediction of the reaction rate of an enzyme was still not possible, in effect defining it as one of today’s continuing ‘grand challenges’ for science. This seems to be a harsh assessment by David Blow in my view, in that qualitatively one can now see directly, for example, that large-substrate enzymes are much slower than small-substrate enzymes. Furthermore, the reaction rate of an enzyme can be deliberately slowed down, or even stopped, by working with a designed mutant of the enzyme, guided by its 3D structure, again illustrating that, if not exactly a prediction of a specific reaction rate, this is a deliberate and successful alteration of the enzyme reaction rate based on crystallo­graphic studies. Overall, then, crystallography has provided a powerful approach to this issue of the relevance of the crystalline state, resulting in a resounding ‘yes’ that these results are relevant to function; this important field has been reviewed by Moffat (2001 ▸). These results, amongst others, thus overcame the objections of the NMR solution-state spectroscopists to the crystallographer’s results in the crystalline state. Weaknesses in the armoury of crystallography remain, such as crystallization conditions, which to a greater or lesser degree take one’s results away from biological functioning conditions. Since those combative times in studies of protein structure, crystallography and NMR have worked in tandem to great effect in understanding structure and dynamics. For example, Fenwick *et al.* (2014 ▸) studied the enzyme dihydrofolate reductase using room-temperature X-ray crystallography and NMR. This study showed agreement between protein backbone and side-chain order parameters derived from NMR relaxation experiments and those calculated from room-temperature single-conformer and multi-conformer models. These results confirmed that the picosecond timescale motions observed in solution were also present in the crystalline state and that quenching took place at cryogenic temperatures. A very recent example of the combined use of NMR and crystallography is reported by Jeganathan *et al.* (2019 ▸). The study of flexibility by NMR is seen as offering new drug-discovery routes in pharmaceutical, health and disease research (Peng, 2009 ▸; Sekhar & Kay, 2019 ▸).

As protein crystallography has delivered ever more 3D structures, getting closer to complexity has proved a new challenge and focus. The accomplishments of virus crystallo­graphy (Rossmann, 2015 ▸) and the crystal structure studies of the ribosome (the Nobel Prize in Chemistry 2009 was awarded to Rama­krishnan, Steitz and Yonath; see https://www.nobelprize.org/prizes/chemistry/2009/summary/) are testimony to the large steps forward to complexity, yielding atomically detailed models of these molecular machines. The ribosome studies in particular required the perfecting of cooling conditions for the crystal so it would yield adequate amounts of X-ray diffraction data on the intense synchrotron beamlines needed to measure these data in a workable period of time.

A theme has steadily emerged in structural biology in the last 20 years, where there is a question about the strict relevance to biology of crystallographic results that are now predominantly based on X-ray diffraction data measured at cryo-temperatures (for an early example pointing this out, see Deacon *et al.*, 1997 ▸). This has been compounded by observations of specific X-ray damage to the crystallized protein (for an early example, see Helliwell, 1988 ▸). Conducting crystallo­graphy at physiological temperatures has then become an objective, but what about radiation damage being much greater at room temperature? Neutron macromolecular crystallography (nMX), whilst pursuing protein structures with experimentally determined protonation states, has also automatically yielded room-temperature structures. Given also that projects succeed in obtaining neutron beam time only when all other methods (X-ray, electron or NMR based) have failed, it is clear that in structural biology there is an improved strategic importance of the nMX method. Indeed, nMX has seen a sustained growth in the number and scope of instruments, and of the software and methods employed, at neutron sources (see, for example, Blakeley & Podjarny, 2018 ▸). Furthermore, the new X-ray lasers yield X-ray diffraction data at room temperature and before radiation damage can kick in: the ‘diffract before the sample is destroyed’ approach (Neutze *et al.*, 2000 ▸). Synchrotron facilities are now also adopting the X-ray laser method of ‘serial femtosecond crystallography’ for the delivery of streams of micrometre-sized samples and thereby are also yielding results at physiological temperatures, albeit not free of radiation damage as at X-ray lasers (see, for example, Schlichting, 2015 ▸). An amazing accomplishment, to my mind, is the room-temperature crystal structure of the 30S ribosome using the Stanford Linac Coherent Light Source (LCLS; Dao *et al.*, 2018 ▸). However, the use of streams of micrometre-sized crystals raises the question of variations in these samples of the biological molecules.

Finally, we must ask precisely what does the term ‘physiological conditions’ mean? The book by D. A. Wharton ‘*Life at the Limits: Organisms in Extreme Environments*’ (Wharton, 2002 ▸) describes examples of life at extremes of temperature, such as thermophilic and hyperthermophilic bacteria, as well as at extremes of pressure at the ocean floor, such as piezophiles; at extremes of pH; at extremes of salt concentration and at extremes of cold.

The most unusual example of life at extreme temperatures that I have heard about are the tardigrades, which are able apparently to survive in extreme environments that would kill almost any other animal (https://en.wikipedia.org/wiki/Tardigrade). Extremes of temperature at which tardigrades can survive, *i.e.* can recover from, include a few minutes at 151°C (304°F), 30 years at −20°C (−4°F), a few days at −200°C (−328°F; 73 K) or a few minutes at −272°C (−458°F; 1 K) (Horikawa, 2012 ▸).

In terms of chemical conditions, an interesting case is heavy water (D_2_O), which can kill animals owing to its toxicity compared with regular water (H_2_O), presumably owing to the kinetic isotope effect, while algae can live in heavy water at a ‘practical growth rate’ (Crespi *et al.*, 1959 ▸). Indeed, isotopically enriched proteins can be prepared in this way using algae and then physical chemistry experiments (Crespi *et al.*, 1959 ▸), such as are routinely used today in neutron protein crystallography of fully deuterated proteins (https://www.ill.eu/users/support-labs-infrastructure/deuteration-laboratory/).

The structures of biological macromolecules in each of these categories show the structural and biochemical adaptations of life that are possible. There is then a variation range of known ‘physiological conditions’. In the laboratory we can explore extremes of any one of the biological structures, be it widening for example the temperature range or the pressure range beyond ‘physiological’. Ultimately any structure that is to be of worth, *i.e.* to be more than an entry in an atlas of structures, has to have predictive value with respect to a biological function or in altering that function, as in my comments on David Blow’s lament above.

## 3. Basics about our three diffraction probes: X-rays, neutrons and electrons   

X-rays are scattered by the electron charge cloud of an atom, *i.e.* in proportion to the atomic number of an atom; H atoms offer the weakest scattering and uranium the strongest. Electrons are scattered by the electrostatic potential surface of the atom’s electrons and nuclear charges. Neutrons are scattered by the nucleus and approximately independently of atomic number, with interesting exceptions such as hydrogen and deuterium, which scatter negatively and positively, respectively. Deuterium scatters neutrons with a strength basically the same as those of carbon, nitrogen and oxygen. Neutrons are also nondestructive, *i.e.* there is no radiation damage. Electron scattering is the strongest and requires the smallest samples. Neutrons are the most weakly scattered and require the largest samples.

These are then the important, core, details in the use of X-rays, electrons or neutrons for structural biology. Indeed, we can ask: is there an ideal probe in structural biology? For protein and nucleic acid crystallography, neutrons are in principle the ideal scattering probe compared with X-rays or electrons as they are free of causing radiation damage. The practical problem for neutrons is that such sources have a weak flux, and first of all solving the structure requires X-rays. The full structure elucidation comes then from combined X-ray and neutron studies. If a crystal cannot be grown then electrons (cryoEM) can be used, but to circumvent beam damage cryo-temperatures have thus far proved to be essential. Electrons are sensitive to hydrogens, which is an advantage over X-rays.

## 4. Nonphysiological crystallization (pH, high salt)   

Our structural biology scientific literature has remarked on worries about nonphysiologically relevant crystallization conditions. Examples include the following.

(i) Cyanomet human haemoglobin crystallized under physiological conditions exhibits the Y quaternary structure (Smith & Simmons, 1994 ▸) and the final remark in the whole paper is the opinion that
  However, our results suggest a review of structure–function correlations in the haemoglobin system, and caution that meaningful structure–function correlations in other systems may require more comparable conditions for crystallographic and functional studies.
(ii) Yibin Lin’s article ‘*What’s happened over the last five years with high-throughput protein crystallization screening?*’ (Lin, 2018 ▸) offered the view that
  Scientists frequently select the protein that is suitable for crystallization but far from the physiological condition.

## 5. Determinations of structure where the variations seen impinge on understanding biological function   

Examples where method-driven variations in the determined structure that impinge on understanding biological function also include, besides the crystallization conditions used, the use of cryo-temperature versus room temperature.

There are a growing number of studies comparing cryo-temperature versus room-temperature crystal structures.

**Figure 1:**
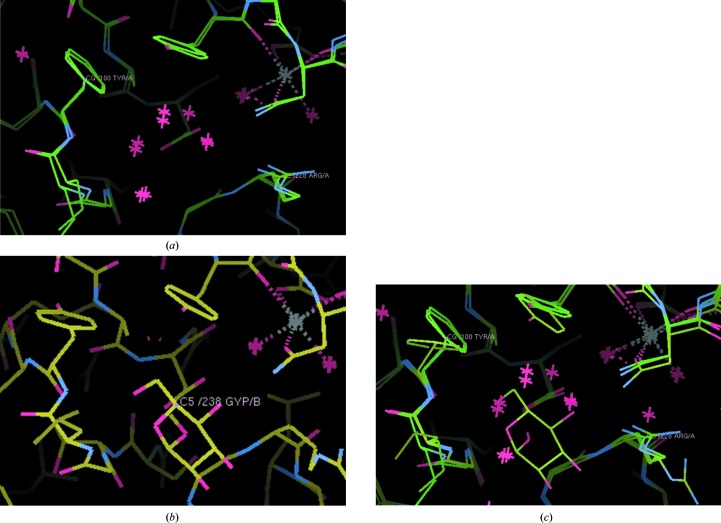
Crystal structure comparisons at the saccharide-binding site of concanavalin A at (*a*) room temperature (PDB entry 1scs; Emmerich *et al.*, 1994 ▸) compared with cryo-temperature (PDB entry 1nls; Deacon *et al.*, 1997 ▸). (*b*) The same view as (*a*) for the glucoside-bound room-temperature crystal structure (PDB entry 1gic; Bradbrook *et al.*, 1998 ▸). (*c*) The view in (*b*) superimposed on the view in (*a*), for which some side-chain adjustments to the glucoside binding and displacement of bound waters are evident and as expected.

An early example was a study of the structure of concanavalin A and its bound solvent determined with small-molecule accuracy at 0.94 Å resolution by Deacon *et al.* (1997 ▸). This cryo-temperature X-ray crystal structure was compared with the room-temperature structure determined by Emmerich *et al.* (1994 ▸) at 1.6 Å resolution. Both were synchrotron X-ray crystal structures and belonged to the same crystallographic space group, *i.e.* crystal packing. The concanavalin A structures compared at the two temperatures showed movements in some amino-acid side chains and in some of the common bound waters. This was not the case in the saccharide-binding site (as demonstrated by directly comparing the cryo-temperature and room-temperature X-ray crystal structures; PDB entries 1nls and 1scs; see Fig. 1 ▸). These cryo-temperature to room-temperature crystal structure comparisons are all described in Section 3.9 of Deacon *et al.* (1997 ▸) both at and away from the saccharide ligand binding site.

That ligand binding can be determined by bound water networks was the conclusion of Darby *et al.* (2019 ▸). They

expected thermodynamic data collected at room temperature to be more interpretable by structural data collected at room temperature rather than those collected at cryogenic temperature. However, our room temperature structures exposed another complication by revealing otherwise hidden, alternative states of the mutated side chain: water positions and occupancies covaried with these alternative states

(see their Fig. 5*c*). Basically, cryo-temperature tipped the amino-acid side chain into a single energy minimum.

Another recent example involving a multi-subunit complex is the study by Young *et al.* (2016 ▸) that compared synchrotron cryo-temperature and X-ray laser room-temperature crystal structures of photosystem II (PSII), in which they saw that the PSII helices had rearranged (see their Fig. 2A). These changes were much larger than the concanavalin A example above. Importantly, the structural layout of the PSII oxygen-evolving complex (OEC) was however not affected by the cryo-temperature.

Overall, these sorts of effects create the anxiety of ‘cryo­artifacts’ (Halle, 2004 ▸).

So, are the cryo-based crystallographic and cryoEM structures that we have carefully determined and validated, archived in our databases and published, at an existential crisis?

Fraser *et al.* (2011 ▸) have argued that there is ‘a bias in structural databases toward smaller, over packed, and unrealistically unique models’ because their ‘analysis suggests that the nearly universal practice of cryocrystallography shifts the intrinsic populations of conformers.’ However, cryoEM has taken the structural biology fight forward, yielding atomic resolution structures of complexes that are too flexible to crystallize essentially; without cryoEM we would have no such structures. In a similar vein, cryocrystallography has allowed the exploitation of high-brilliance third-generation synchrotron-radiation X-ray sources, otherwise room-temperature (down to say 4°C) data collection would have not been feasible owing to radiation damage and thermal effects (Helliwell, 1984 ▸). So, many more biological crystal structures have been determined, and of larger molecular weights, as a result of the exploitation of high-brilliance third-generation synchrotron-radiation sources in macromolecular crystallography (Abad-Zapatero, 2012 ▸; Jiang & Sweet, 2004 ▸). In any case, in many situations accompanying a new structure, be it from cryocrystallography or cryoEM, there is corroborating research such as assaying function at room temperature, and this is an obligatory requirement for publishing such a study. As an example, in unravelling the structural chemistry of the coloration mechanism of lobster crustacyanin, the methods of biochemistry and biological cryocrystallography with UV–Vis spectroscopy and liquid solution X-ray scattering at room temperature, as well as electron microscopy, were applied to study the molecular basis of the colouration in lobster shell (Chayen *et al.*, 2003 ▸). In this study, in a nutshell, the crystals and solutions of crustacyanin studied at room and at cryo-temperature remained blue in colour.

A further remark to conclude this section is to mention that crystals grown *in vivo*, although very tiny, can now yield room-temperature crystal structures through the use of X-ray laser synchrotron beams. A survey has been made of this topic by Duszenko *et al.* (2015 ▸).

## 6. Extremes of pressure and impact on protein crystal structure   

There has been a growth in protein crystallography studies at high pressure. These nicely document changes under this particular extreme. There are piezophiles, organisms that survive on the ocean floor, but these are still exposed to relatively modest pressures compared with those that can be studied with protein crystallography. A strict definition of a piezophile is an organism whose growth rate is maximal at higher pressure. The current record for the highest hydrostatic pressure at which growth has been observed is 130 MPa (1283 atm, 18 855 pounds per square inch) by the archaeon *Thermococcus piezophilus* (Dalmasso *et al.*, 2016 ▸).

Crystallographic laboratories that have been active in this field include those led by Roger Fourme, Sol Gruner and Nobuhisa Watanabe. Some examples of their research results in this area are described below as illustrations of what can be learnt from high-pressure protein crystallography.

The Fourme laboratory studied the adaptation of the base-paired double helix to extreme hydrostatic pressure (Girard *et al.*, 2007 ▸). Four complete diffraction data sets at high resolution (1.60–1.65 Å) were recorded at ambient pressure, 0.55, 1.04 and 1.39 GPa, and the crystal structures were fully refined. They found that the average base-pair step varied from 2.92 to 2.73 Å, but that the transversal compressibility was negligible. The molecule reacted under high pressure basically as a molecular spring but, during compression, the geometry of the Watson–Crick base pairings, which carry the genetic information, was preserved. They remark on the importance of these results as follows.

Accordingly, the double-helix topology is remarkably adapted to high pressure and the adaptation of such architectures to harsh conditions may have played an important role at the prebiotic stage and in the first steps of the emergence of life.

[Since such high pressures do not exist in nature, one assumes that ‘harsh conditions’ refers to the chemical conditions of the pre-biotic time.]

The Gruner laboratory probed substates in sperm whale myoglobin using high-pressure crystallography (Urayama *et al.*, 2002 ▸). There are displacements in the F-helix, AB-loop and CD-loop regions. This study also made structure-to-structure comparisons not only between pressures but also between room temperature and cryo-temperature, and by varying the pH. It was under high pressure at room temperature that the F helix slid along its axis and moved towards the E helix.

The Watanabe laboratory used high-pressure protein crystallography and thereby explained why one of the active-site residues of lysozyme, Glu35, has a high p*K*_a_ value (Yamada *et al.*, 2015 ▸). They found different conformations at three pressures; at 0.1 and 950 MPa Glu35 showed quite different side-chain conformations, while at 890 MPa a split between the two conformations was observed. Based on their crystal structures they then used two different protonation-prediction algorithms, which estimated the p*K*_a_ value of Glu35 in each conformation as 6.4 and 4.5, which was important for its role as a general acid catalyst. An evaluation review of the prediction of ionizable amino-acid protonation states in proteins by three different software packages versus crystallographic experiment (X-ray and neutron studies) and NMR protonation titration studies is available; this showed that the average likelihood of a correct protonation prediction for glutamic acid was 63% (Fisher *et al.*, 2009 ▸).

Finally, as Keedy (2019 ▸) has correctly argued, I think that ‘by combining different perturbations such as temperature, pressure, pH *etc.* families of models [can] map how different parts of a structure collectively respond to stimuli’ or indeed better reflect the likely physiological situation.

## 7. Conclusions   

In summary, the physical methods of crystallography, microscopy and spectroscopy continue to strive for, and clearly deliver, biologically relevant results. From these studies, the prediction of aspects of biological function from atomic structures is possible and is also physiologically relevant. Future directions will surely see an increase in the diversity of structures under a variety of measurement conditions deposited in the Protein Data Bank (PDB). The PDB guides users of any given deposition by its slider diagram of the quality of a structure. This is dominated by the diffraction resolution, as an example for crystallography, which overall determines (*i.e.* improves) each subsidiary metric in the PDB slider diagram. Clearly, I advocate in this feature article that an additional overarching metric of physiological relevance be adopted in the PDB for any given deposition, for example, room temperature is better than cryo-temperature, crystallization conditions closer to rather than further from *in vivo* are preferred, the structure determined is corroborated using functional assays *etc*. That said, X-ray laser and neutron crystal structures deliver physiological temperature results, free of radiation damage, as a matter of course. Synchrotron beamlines are also offering the room-temperature serial femto­second crystallography option such as at MAX IV (Marjorlein Thunissen, personal communication). Basically, these days we can have structural biology at standard temperature and pressure and under as close to physiological conditions as possible.

As well as the above rather general remarks, there are then the following specific questions.

(i) Are the general observations raised here with regard to cryocooling actually significant (in the common sense of the word) for most structures? Whilst many cases of radiation damage have been demonstrated, including metal oxidation or reduction, disulfide-bond breakage or deamination, they are not all, or necessarily, biologically significant. Halle (2004 ▸), however, does document functionally significant situations affected by cryo-temperature.
(ii) More recent advances using quantum mechanics-based refinement (see, for example, Goerigk *et al.*, 2014 ▸), rather than the currently common simple spherical density-based models, may well allow some of the ‘nonphysiological’ experimental limitations to be overcome. The quantum mechanics-based approach may provide the ability to ‘change’ the conditions of an experiment afterwards if the theoretical calculations are sufficiently accurate and if the computer power is good enough, even for very large complexes such as those now routinely studied by cryoEM.
(iii) While we know the temperature optimum for most organisms, the interior of a cell is not a dilute solution and is not uniform. How can we quantify this sort of natural variation, *i.e.* as distinct from the variations in our results from our various investigative methods?
(iv) In practical terms the freezing of a single particle for cryoEM is not the same outcome necessarily as that arising from cooling a 1 µm crystal for a serial femtosecond crystallography X-ray laser experiment or a, say, 50 µm crystal for a third-generation synchrotron-radiation macromolecular crystallo­graphy experiment. If the new generation of X-ray lasers do realize the single protein-molecule structure at room temperature (Neutze *et al.*, 2000 ▸), and this objective is still in fact on the road maps of these X-ray facilities, then the comparison evaluations of structures determined by cryoEM and at room temperature using such new X-ray lasers would come much closer.
